# Efficacy and safety of 0.05% ingenol mebutate in the treatment of basal cell carcinoma: A prospective study

**DOI:** 10.1002/ski2.150

**Published:** 2022-08-10

**Authors:** Marine Velin, Nathalie Cardot‐Leccia, Anne‐Claire Cathelineau, Luc Duteil, Catherine Queille‐Roussel, Thierry Passeron, Philippe Bahadoran

**Affiliations:** ^1^ Department of Dermatology Université Nice Côte d’Azur CHU Nice Nice France; ^2^ Department of Pathology Université Nice Côte d’Azur CHU Nice Nice France; ^3^ CPCAD (Center of Clinical Pharmacology Applied to Dermatology) Archet 2 Hospital Nice France; ^4^ INSERM U1065 Centre Méditerranéen de Médecine Moléculaire Université Nice Côte d’Azur Nice France

## Abstract

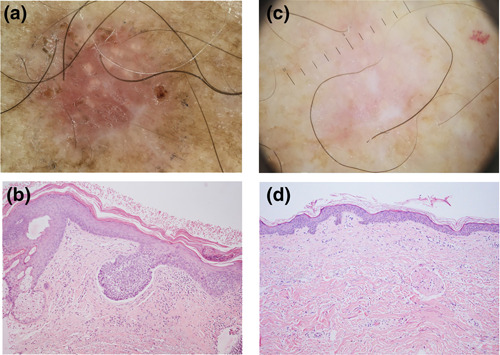

Dear Editor,

Basal cell carcinoma (BCC) is the most common type of skin cancer.^1^ Surgery is considered to be the gold standard for BCC treatment, although low‐risk BCCs can be treated with non‐surgical topical methods such as imiquimod, 5‐fluorouracil (5‐FU), and photodynamic therapy (PDT).^2^ The use of imiquimod and 5‐FU can be limited, however, by the fact that patients have to treat themselves for several weeks, while the use of PDT is limited by the availability of the equipment. Therefore, there is a need to find additional topical treatments for BCC. Ingenol mebutate (IM, Picato®, LEO Pharma), a drug approved in 2012 by the FDA and EMA for short‐term treatment of actinic keratosis (AK), at a concentration of 0.015% or 0.05% pending on location, could provide a faster and easier topical therapy for BCC. Data regarding BCC treatment with IM are limited, however.^3^, ^4^

We therefore conducted a prospective, exploratory, uncontrolled, single‐site study in the Dermatology Department of Nice University Hospital to investigate the efficacy and safety of one or two courses of 0.05% IM in superficial and nodular BCC (‘PICABAS’ study). However, during the course of the study the European marketing authorization of IM was withdrawn in 2020 at the request of LEO Pharma after a review by the EMA that concluded that IM may increase the risk of cutaneous squamous cell carcinoma (SCC) in patients treated for AK.^5^ Eligible participants were 18 years of age or older, with at least one histologically verified primary superficial or nodular BCC on the trunk or limbs. Ingenol mebutate gel (0.05%) was applied once daily for two consecutive days. Basal cell carcinoma responses were assessed on day 90 after treatment. If the tumor was no longer clinically visible (clinical examination including dermoscopy analysis), a three‐mm biopsy was made in order to confirm histological clearance. If the lesion was still clinically visible or if the biopsy revealed residual BCC, a second treatment course was performed under the same modalities. Local adverse events were assessed at each cycle based on the local skin reaction (LSR) score. The primary outcome was the proportion of complete responses, defined as complete clinical and histological clearance assessed 3 months after one or two cycles of IM treatment.

We performed a modified Intent‐To‐Treat (mITT) analysis, defined as all BCCs which met the inclusion criteria, received at least one application of IM, and which underwent at least one post‐treatment evaluation. We also did a Per‐Protocol (PP) analysis, including all BCCs which met the inclusion criteria, received the full study treatment (one or two cycles if needed according to the study protocol), and which did not have any major deviation. The mITT and the PP population comprised 39 and 28 BCCs, respectively. Basal cell carcinomas were located on the trunk (52.6%), arm (28.9%), or leg (18.4%) The mean diameter of the BCCs was 1.1 cm (SD 0.38) and the median diameter was 1 cm (range 0.6–2.5). There were 20 sBCC (52.6%) and 18 nBCC (47.4%). The mean histological thickness was 0.67 mm (SD 0.61), and the median thickness was 0.4 mm (0.1–2.6). The complete response rate for sBCC was between 50% (11/20, mITT) and 62.5% (10/16, PP) (Figure 1). For nBCC, the complete response rate was between 5.6% (1/18, mITT) and 8.3% (1/12, PP). The multivariate analysis showed that superficial BCC histotype was a significant independent prognostic factor of complete response to IM (OR 14.89, *p* = 0.036). The median LSR score was 7.0 (range 1–13) for the first treatment course and 6.5 (range 0.0–15.0) for the second, as reported for AK treatment. Following the EMA review concluding the IM may increase the risk of cutaneous SCC in patients treated for AK, we conducted an additional safety follow‐up 14 months after the last application of IM. No occurrence of SCC was noted in 27 BCC cases treated with IM. However this is not a long enough period to conclude about the safety of IM in patients treated for BCC.

**FIGURE 1:**
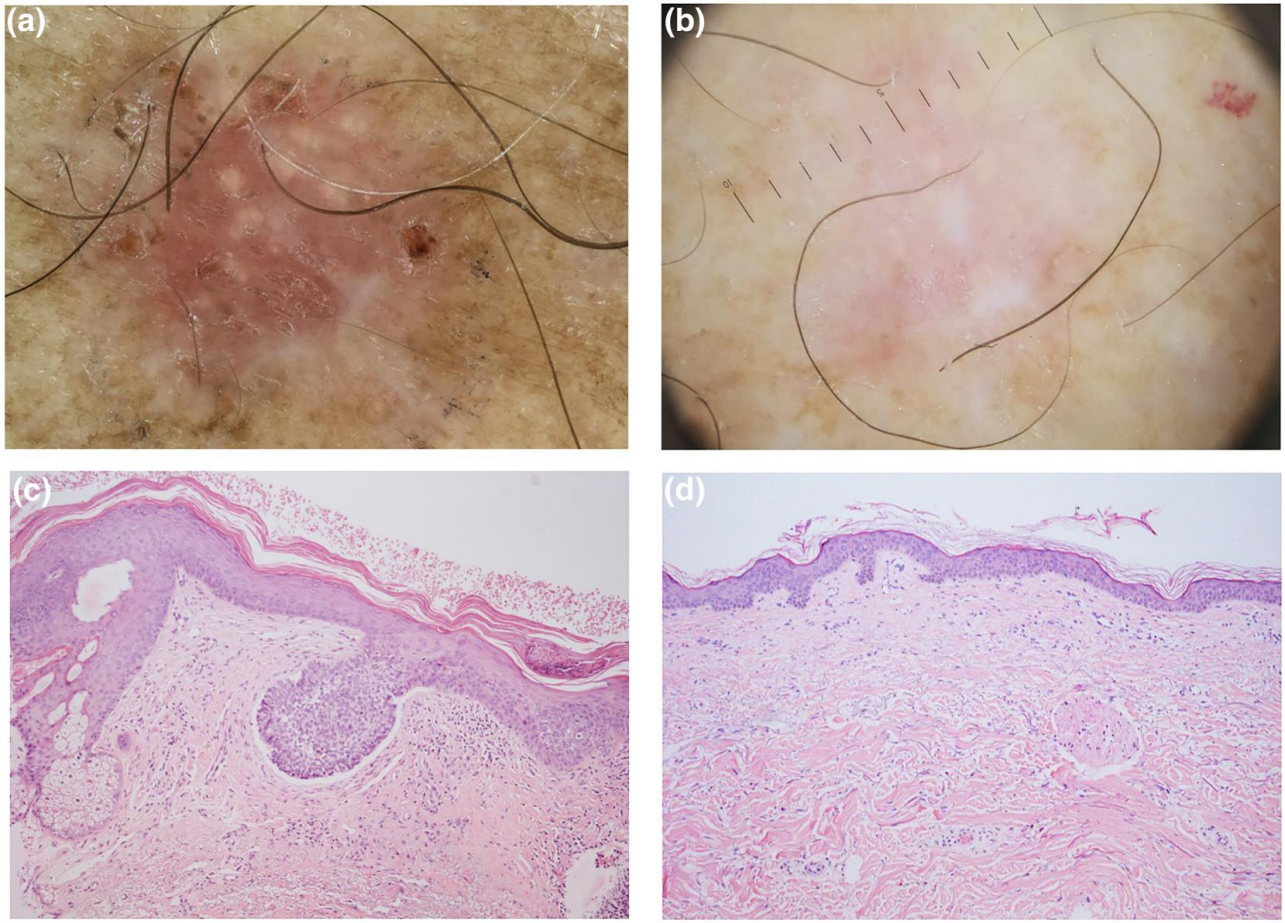
Complete response of a superficial basal cell carcinoma (BCC) of the trunk after two cycles of 0.05% ingenol mebutate. Dermoscopic and histological aspects before (a, b) and after (c, d) treatment

In conclusion, ingenol mebutate does not appear to be a useful addition to the existing treatment options for low‐risk superficial BCC, and cannot be recommended for the treatment of low risk nodular BCC.

## AUTHOR CONTRIBUTION

**Philippe Bahadoran**: Conceptualization (Equal); Investigation (Equal); Methodology (Equal); Supervision (Equal); Validation (Equal); Writing – original draft (Equal); Writing – review & editing (Equal). **Marine Velin**: Conceptualization (Equal); Investigation (Equal); Methodology (Equal); Validation (Equal); Writing – original draft (Equal). **Nathalie Cardot‐Leccia**: Conceptualization (Equal); Investigation (Equal); Methodology (Equal); Validation (Equal); Writing – original draft (Equal). **Anne‐Claire Cathelineau**: Conceptualization (Equal); Investigation (Equal); Methodology (Equal); Validation (Equal); Writing – original draft (Equal). **Luc Duteil**: Conceptualization (Equal); Investigation (Equal); Methodology (Equal); Validation (Equal); Writing – original draft (Equal). **Catherine Queille‐Roussel**: Conceptualization (Equal); Investigation (Equal); Methodology (Equal); Validation (Equal); Writing – original draft (Equal). **Thierry Passeron**: Conceptualization (Equal); Investigation (Equal); Methodology (Equal); Validation (Equal); Writing – original draft (Equal).

## FUNDING INFORMATION

Leo Pharma.

## CONFLICTS OF INTEREST

Philippe Bahadoran received consulting fees from Leo Pharma.

## ETHICS STATEMENT

People Protection Committee of Ile‐de‐France VIII, N° 2017‐004608‐22.

## CLINICALTRIALS.GOV REGISTRY

NCT03546166.

## Data Availability

Data available on request from the authors.
